# Adaptation of anaerobic cultures of *E* *scherichia coli* K‐12 in response to environmental trimethylamine‐N‐oxide

**DOI:** 10.1111/1462-2920.12726

**Published:** 2015-02-03

**Authors:** Katie J. Denby, Matthew D. Rolfe, Ellen Crick, Guido Sanguinetti, Robert K. Poole, Jeffrey Green

**Affiliations:** ^1^ The Krebs InstituteDepartment of Molecular Biology and BiotechnologyUniversity of SheffieldSheffieldS10 2TNUK; ^2^ School of InformaticsInformatics Forum10 Crichton StreetEdinburghEH8 9ABUK

## Abstract

Systematic analyses of transcriptional and metabolic changes occurring when *E* *scherichia coli* K‐12 switches from fermentative growth to anaerobic respiratory growth with trimethylamine‐N‐oxide (TMAO) as the terminal electron acceptor revealed: (i) the induction of *torCAD*, but not genes encoding alternative TMAO reductases; (ii) transient expression of *frmRAB*, encoding formaldehyde dehydrogenase; and (iii) downregulation of copper resistance genes. Simultaneous inference of 167 transcription factor (TF) activities implied that transcriptional re‐programming was mediated by 20 TFs, including the transient inactivation of the two‐component system ArcBA; a prediction validated by direct measurement of phosphorylated ArcA. Induction of *frmRAB*, detection of dimethylamine in culture medium and formaldehyde production when cell‐free extracts were incubated with TMAO suggested the presence of TMAO demethylase activity. Accordingly, the viability of an *frmRAB* mutant was compromised upon exposure to TMAO. Downregulation of genes involved in copper resistance could be accounted for by TMAO inhibition of Cu(II) reduction. The simplest interpretation of the data is that during adaptation to the presence of environmental TMAO, anaerobic fermentative cultures of *E* *. coli* respond by activating the TorTSR regulatory system with consequent induction of TMAO reductase activity, resulting in net oxidation of menaquinone and inhibition of Cu(II) reduction, responses that are sensed by ArcBA and CusRS respectively.

## Introduction

Trimethylamine‐N‐oxide (TMAO) is an osmolyte in marine organisms and is often found in anaerobic environments where it is used as a terminal electron acceptor, being reduced to trimethylamine (TMA) to support the growth of enteric bacteria (Barrett and Kwan, 1985). TMA, generated from metabolism of dietary phosphatidylcholine, L‐carnitine and TMAO (from fish) by human gut microflora is transferred to the liver, where, after oxidation to TMAO by flavin monooxygenases, it promotes cardiovascular disease (Wang *et al*., 2011; Koeth *et al*., 2013). *Escherichia coli* K‐12 is a metabolically versatile enteric bacterium that is able to grow in the presence and absence of O_2_. Aerobic respiration, anaerobic respiration and fermentation represent its three major metabolic modes (Bock and Sawers, 1996; Gennis and Stewart, 1996; Guest *et al*., 1996). Anaerobic respiration with TMAO as the terminal electron acceptor conserves less energy than aerobic respiration but is more efficient than fermentation (Guest *et al*., 1996). TMAO is reduced to TMA by the enzyme TMAO reductase, encoded by the *torCAD* operon (Méjean *et al*., 1994). The expression of *torCAD* is controlled by a modified two‐component system comprised of three proteins: TorT, S and R. TorT is a periplasmic TMAO‐binding protein (Baraquet *et al*., 2006) that interacts with the membrane protein TorS to form a sensory complex capable of transmitting the signal (TMAO) across the cytoplasmic membrane to alter the behaviour of the regulator TorR (Moore and Hendrickson, 2012). Upon stimulation the TorS protein phosphorylates the regulator TorR, which then activates transcription of the *torCAD* operon (Simon *et al*., 1994; 1995). In the absence of TMAO, TorS acts as a TorR∼P phosphatase to ensure that TMAO reductase is not produced in the absence of its substrate (Ansaldi *et al*., 2001). The TMAO reductase subunit (TorA) has a molybdopyranopterin cofactor and is located in the periplasm. Electrons from reduced quinones (menaquinol and dimethylmenaquinol; Wissenbach *et al*., 1992) are transferred to TorA via the membrane‐associated penta‐heme cytochrome TorC (McCrindle *et al*., 2005). Thus, reduction of TMAO can be coupled to the action of various dehydrogenases that reduce the quinone pool and in this way energy is conserved chemiosmotically. Unlike other alternative electron acceptors that are used by *E. coli*, TMAO respiration has been reported to occur in the presence of the preferred electron acceptor O_2_ (Ansaldi *et al*., 2007).

Thus, there is a body of knowledge covering the regulation and operation of the *E. coli* TMAO respiratory chain, but crucially there is almost no understanding of the dynamic adaptive processes that occur during the transition from anaerobic fermentative to TMAO respiratory growth. Here, glucose‐limited chemostat cultures have been used to systematically study the effects of perturbing anaerobic fermentative cultures of *E. coli* K‐12 by addition of the terminal electron acceptor TMAO using transcript profiling, metabolite and biochemical measurements combined with probabilistic modeling of transcription factor (TF) activities to obtain a deeper understanding of the dynamics of the adaptive process.

## Results and discussion

### Analysis of over‐metabolite production by anaerobic cultures of *E* *. coli* K‐12 in the absence and presence of TMAO

Glucose‐limited anaerobic fermentative growth of *E. coli* can be described by Eq. (1) and (2):

(1) $$C_{6}H_{12}O_{6}+H_{2}O\to CH_{3}COOH+CH_{3}CH_{2}OH+2\;HCOOH$$

(2) $$HCOOH\to CO_{2}+H_{2}$$

In addition, other minor fermentation products (over‐metabolites) such as lactate and succinate are produced. Anaerobic steady‐state chemostat cultures (20 mM glucose‐limited medium, dilution rate 0.2 h) of *E. coli* K‐12 MG1655 were established, and measurement of over‐metabolites present in the culture medium at steady state showed the presence of acetate, ethanol, formate, succinate, fumarate, lactate and orotate, consistent with anaerobic fermentative growth during which ∼40% of the formate was converted to CO_2_ and H_2_ by formate hydrogen‐lyase (Table 1; Eq. (2)). The anaerobic fermentative steady state was perturbed by the introduction of TMAO to both the cultures and the medium feed. Fully anaerobic respiratory growth on glucose with TMAO as the electron acceptor is described by Eq. (3).

(3) C6H12O+6 4 (CH3)3 NO→2 CH3COOH+4 (CH3)3 NH2+ 2 CO2+2 H2O

**Table 1:** Measurements of extracellular metabolites during transition of *E* *. coli* MG1655 from anaerobic fermentative growth to TMAO‐respiratory/fermentative growth

Time after addition of TMAO (min)	TMAO (mM)	TMA (mM)	DMA (mM)	Acetate (mM)	Formate (mM)	Ethanol (mM)^a^	Succinate (mM)	Lactate (mM)	Fumarate (mM)	Orotate (mM)	Biomass (mg cell dry weight ml^−1^)
0	ND	ND	ND	13.7 ± 0.6	13.6 ± 2.7	10.9 ± 0.2	3.9 ± 0.2	0.08 ± 0.01	0.03 ± 0.01	0.16 ± 0.01	0.29 ± 0.07
2	45.8 ± 1.1	0.4 ± 0.1	0.08 ± 0.01	13.6 ± 0.7	11.0 ± 2.8	10.7 ± 0.1	3.7 ± 0.2	0.05 ± 0.01	0.02 ± 0.01	0.13 ± 0.02	
5	46.0.5 ± 1.2	0.5 ± 0.1	0.09 ± 0.01	13.7 ± 0.7	10.9 ± 2.8	10.7 ± 0.1	3.7 ± 0.2	0.06 ± 0.01	0.03 ± 0.01	0.11 ± 0.01	
10	45.0 ± 1.2	0.8 ± 0.1	0.10 ± 0.01	13.5 ± 0.8	10.6 ± 2.8	10.4 ± 0.1	3.6 ± 0.2	0.05 ± 0.01	0.03 ± 0.01	0.12 ± 0.01	
15	44.5 ± 1.3	1.1 ± 0.1	0.12 ± 0.02	13.4 ± 0.9	10.6 ± 3.0	10.2 ± 0.1	3.6 ± 0.2	0.05 ± 0.01	0.04 ± 0.01	0.13 ± 0.02	
20	43.2 ± 2.0	1.4 ± 0.1	0.12 ± 0.02	13.2 ± 1.0	10.5 ± 3.0	10.0 ± 0.2	3.4 ± 0.2	0.06 ± 0.01	0.05 ± 0.01	0.13 ± 0.02	
60	40.4 ± 2.3	4.2 ± 0.5	0.23 ± 0.01	13.6 ± 1.0	11.0 ± 3.0	10.7 ± 1.1	3.1 ± 0.2	0.07 ± 0.01	0.08 ± 0.03	0.12 ± 0.04	0.44 ± 0.08
1440	ND	45.8 ± 1.4	0.15 ± 0.03	24.5 ± 1.2	6.7 ± 1.4	2.3 ± 0.4	1.5 ± 0.6	ND	0.05 ± 0.01	0.37 ± 0.02	0.61 ± 0.04

a The values reported for ethanol are corrected to account for losses to the gas phase.

Data are the mean values ± standard deviation (*n* = 3). ND, not detected.

In the experiments reported here, 46 mM TMAO was introduced into the system. Under these conditions, a mixed anaerobic respiratory and fermentative metabolism (hereafter referred to as TMAO‐respiratory/fermentative growth) was expected and is described by Eq. (4).

(4) $$\begin{array}{l} C_{6}H_{12}O_{6}+2.4\;{\left(CH_{3}\right)}_{3}\;NO\to 1.6\;CH_{3}COOH+\\ \;0.4\;CH_{3}CH_{2}OH+2.4\;{\left(CH_{3}\right)}_{3}\;NH_{2}+\\ \;0.5\;HCOOH+1.5CO_{2}+0.76\;H_{2}O \end{array}$$

**Figure 1:**
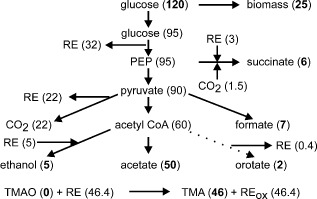
Metabolic map of the TMAO‐respiratory/fermentative steady state. The carbon source glucose provides 120 atoms of carbon to the system, 25 of which are incorporated into biomass based on ∼50% of cdw is carbon (Heldal *et al*., 1985). Therefore, 95 carbon atoms enter glycolysis generating 32 reducing equivalents (RE) in the production of phosphoenolpyruvate (PEP). A small amount of PEP is converted to succinate by reaction with CO_2_ (six, carbon atoms) concomitantly re‐oxidizing three RE (Table 1). The remainder of the PEP (90 carbon atoms) is converted to pyruvate, 26% by pyruvate formate‐lyase and 74% by the pyruvate dehydrogenase complex, the latter generating 22 RE. The majority of the resulting acetyl CoA (60 carbon atoms) is converted to acetate (50 carbon atoms) along with small amounts of ethanol (five carbon atoms; re‐oxidizing five RE) and orotate (two carbon atoms; generating 0.4 RE) (Table 1). In this scheme, a total of 46.4 RE are created and re‐oxidized (RE_ox_) by reduction of TMAO to 46 molecular equivalents of TMA (Table 1). Bold numbers in parentheses are measured quantities (Table 1); those shown in normal font are estimates.

For 60 min following the addition of TMAO, the concentration of fumarate increased, whereas the concentrations of succinate and formate decreased (Table 1). During the same period, the concentration of TMAO decreased from ∼46 mM to ∼40 mM. After 1440 min, the new TMAO‐respiratory/fermentative steady state was established and TMAO was undetectable, whereas TMA reached ∼46 mM. The external milieu of the new steady state had higher acetate and orotate, but lower succinate and ethanol concentrations than those observed under fermentative conditions (Table 1). The physiological changes characterized by these metabolite measurements were accompanied by an increase in biomass (Table 1). The biomass supported by the TMAO‐respiratory/fermentative steady state was ∼twofold greater than that under fermentative conditions (0.61 g l^−1^ compared 0.29 g l^−1^). The dry weight of an *E. coli* cell is 0.28 pg (Neidhardt *et al*., 1990). Therefore, this increase in biomass was equivalent to an additional ∼1.2 × 10^12^ bacteria l^−1^. An electron transport chain consisting of NADH dehydrogenase I and TMAO reductase contributes 6 H^+^ to the proton motive force per NADH molecule oxidized (Keseler *et al*., 2013). For an H^+^/ATP ratio of 3.3 (Futai *et al*., 2012), 1.8 ATP molecules are produced for every TMAO molecule reduced, which equates to the production of 5 × 10^22^ molecules of ATP. The Y_ATP_ value for *E. coli* growing under anaerobic conditions at a dilution rate of 0.2 h is ∼5 g cell dry weight (cdw) per mol of ATP (Hempfling and Mainzer, 1975). Assuming that glucose fermentation yieds 3 ATP per glucose molecule, this value is consistent with the cdw of the initial anaerobic steady‐state cultures analysed here (60 mmol ATP from 20 mmol glucose yielding 0.29 g cdw is equivalent to Y_ATP_ = 4.8 g cdw per mol of ATP (Table 1). Therefore, the synthesis of an *E. coli* cell requires ∼40 × 10^9^ ATP molecules, and hence the additional ATP gained from respiration with TMAO as the terminal electron acceptor accounts for the synthesis of an additional ∼1.3 × 10^12^ cells. Thus, the metabolic map shown in Fig. 1 accurately captures the physiological state of the TMAO‐respiratory/fermentative steady state.

### The transcriptional response of *E* *. coli* K‐12 during adaptation to TMAO‐respiratory conditions

Transcript profiles were obtained for *E. coli* K‐12 cultured under anaerobic fermentative conditions and then 2, 5, 10, 15, 20 and 60 min after the addition of the electron acceptor TMAO and under the new TMAO‐respiratory/fermentative steady state (1440 min). For this analysis, an operon was deemed to be significantly regulated if, at one or more of the sampling points, transcript abundance was ≥ threefold greater or smaller after the addition of TMAO compared with the initial anaerobic fermentative steady state (Table 2). In total, 34 operons were judged to be significantly regulated (29 were upregulated and 5 were downregulated).

**Table 2:** Transcripts that are altered in abundance by ≥ threefold in response to the addition of TMAO to anaerobic fermentative steady‐state cultures of *E* *. coli* K‐12 MG1655

Operon^a^	Product	Fold‐change in abundance relative to the anaerobic fermentation steady state^b^							Regulatory proteins^c^
		2 min	5 min	10 min	15 min	20 min	60 min	TMAO respiratory steady state	
Predominantly upregulated operons									
(A) Terminal recuctase/oxidases									
*cyoA‐E*	Cytochrome *bo*′ terminal oxidase	2.5	3.8	6.4	3.3	3.2	2.2	1.1	ArcA (−), Cra (−), CRP (+), FNR (−), Fur (−), GadE (+), PdhR (−)
*torCAD*	TMAO reductase	4.4	6.1	11.6	7.9	7.6	7.6	3.5	NarL (−) TorR (+)
(B) Carbon metabolism									
*acs‐yjcH‐actP*	Acetyl‐CoA synthetase, inner membrane protein, acetate transporter	1.7	3.3	6.1	3.1	1.8	1.0	1.2	CRP (+), Fis (−), IHF (−)
*frmRAB*	Transcription regulator, formaldehyde dehydrogenase, *S*‐formylglutathione hydrolase	2.4	4.6	4.9	6.1	10.9	9.5	1.3	FrmR (−)
*glpABC*	Anaerobic glycerol‐3‐phosphate dehydrogenase	2.1	3.5	2.1	1.3	−1.2	−1.4	1.0	ArcA (−), CRP (+), Fis (+), FlhDC (+), FNR (+), GlpR (−)
*glpD*	Aerobic glycerol‐3‐phosphate dehydrogenase	2.9	4.6	2.8	1.6	1.2	1.1	1.2	ArcA (−), CRP (+), GlpR (−)
*glpFK*	Glycerol channel and glycerol kinase	2.2	3.8	3.4	1.2	−1.3	−1.3	1.3	CRP (+), GlpR (−)
*pdhR‐aceEF‐lpd*	PdhR regulator, pyruvate dehydrogense complex	1.6	1.7	2.3	3.1	2.7	1.6	1.0	ArcA (−), Cra (−), CRP (+), Fis (+), FNR (±), Fur (±), NsrR (−), PdhR (−)
*sdhCDAB‐sucA‐D*	Succinate dehydrogenase, 2‐oxo‐glutarate dehydrogenase	2.3	3.6	3.6	2.4	1.6	1.3	1.2	ArcA (±), CRP (+), FNR (−), Fur (+)
(C) Methionine metabolism									
*metBL*	*O*‐Succinylhomoserine lyase, aspartate kinase	1.2	−1.3	1.2	2.4	5.6	1.2	1.2	MetJ (−), PhoP (+), YjiE (+)
*metF*	5, 10‐Methylenetetrahydrofolate reductase	−1.1	−1.5	−1.2	1.5	3.8	1.1	1.1	MetJ (−)
*metR*	Methionine regulator	1.0	−1.3	−1.2	1.4	3.2	1.1	1.0	MetJ (−), MetR (−)
*mmuPM*	*S*‐Methyl‐L‐methionine transport	1.1	1.1	1.1	1.9	3.5	1.1	1.1	
*sbp*	Sulfate‐binding proten	−1.1	1.0	1.0	1.8	3.6	1.7	−1.2	
*ybdL*	Methionine aminotransferase	1.1	−1.6	1.0	1.8	4.0	1.0	1.1	
(D) Transport proteins									
*cstA*	Peptide transport	2.2	4.0	3.6	1.4	1.1	1.1	1.6	CRP (+)
*dctA*	C4‐dicarboxylate/orotate : proton symport	2.1	3.8	4.3	1.8	1.6	1.3	1.6	ArcA (−), CRP (+), DcuR (+)
*mglBAC*	Galactose transport	2.1	2.5	3.7	1.1	1.0	−1.2	2.0	Crp (+), GalR (−), GalS (−)
*ugpBAECQ*	Glycerol‐3‐phosphate transport	1.8	3.3	2.4	1.5	1.2	1.2	1.2	PhoB (+)
(E) Motility and chemotaxis									
*flgAMN*	Flagella biosynthesis	1.0	1.1	−1.1	1.1	1.1	−1.1	3.0	FlhDC (+)
*flgB‐J*	Flagella proteins	−1.1	−1.2	−1.1	−1.2	−1.1	1.0	3.3	FlhDC (+)
*fliAZY*	Flagella regulation and biosynthesis	−1.1	−1.1	−1.3	−1.1	−1.1	1.1	4.0	FlhDC (+), H‐NS (+), MarA (−), NsrR (−)
*motB‐cheAW*	Flagella and chemotaxis proteins	1.1	−1.1	1.0	1.1	1.0	1.0	3.2	CpxR (−)
*tar‐tap‐cheRBYZ*	Chemotaxis proteins	1.0	1.3	1.5	1.1	−1.1	1.0	3.1	FNR (+)
(F) Miscellaneous									
*betIBA*	Betaine aldehyde dehydrogenase and regulatory protein	1.8	3.3	2.5	1.9	1.6	1.4	1.0	ArcA (−), BetI (−)
*bsmA*	Conserved protein	1.7	3.0	1.4	1.0	−1.2	1.0	1.0	
*glcC*	Glycolate regulator	1.8	3.9	2.3	1.4	1.1	1.0	1.0	Cra (−), CRP (+), Fis (−), GlcC, (−)
*putA*	Fused transcriptional repressor‐proline dehydrogenase	1.6	2.8	5.5	2.2	2.2	1.7	1.2	BasR (−), MarA (+), PutA (−)
*ybdH*	Predicted oxidoreductase	1.3	1.2	1.3	1.9	3.0	1.1	1.2	
(G) Predominantly downregulated operons									
*borD*	Predicted lipoprotein	−1.8	−3.0	−1.4	1.5	1.8	1.2	1.1	PhoP (+)
*cusCFBA*	Copper efflux	−1.1	−2.2	−3.3	−2.5	−3.0	−2.9	1.0	CusR (+)
*manXYZ*	Mannose PTS permease	−1.8	−1.4	−3.7	−5.0	−3.0	−2.3	−1.1	CRP (+), Cra (−), DgsA (−), NagC (−)
*ompF*	Outer membrane protein	1.0	−2.4	−3.1	−2.4	−1.3	1.1	1.7	CpxR (−), CRP (+), EnvY (+), Fur (+), IHF (±), OmpR (±), RstA (−)
*thrABC*	Threonine and homoserine biosynthesis	−1.6	−1.6	−2.4	−3.2	−2.3	1.0	−1.5	DksA (+)

a The data shown are for the first gene in the operon unless indicated in bold type.

b Numbers are fold increase or decrease (by at least threefold, *P* ≤ 0.05, at one or more sampling points) in transcript abundance after introduction of TMAO.

c Regulatory proteins are indicated (−) negative regulation, (+) positive regulation, (±) dual regulation.

PTS, phosphotransferase system.

The upregulated transcripts could be classified into six groups based on the functions of the encoded proteins (Table 2). Unsurprisingly, within 2 min of the addition of TMAO (46 mM) to the system the *torCAD* (TMAO reductase) operon was induced (Table 2; group A), indicating that the presence of the electron acceptor had been perceived and a transcriptional response had been initiated. The metabolite measurements indicated that TMAO reduction to TMA was initiated within 2 min (Table 1). The *torCAD* operon transcript exhibited maximal abundance (11.6‐fold greater than in the anaerobic fermentative steady state) 10 min after initial exposure of the culture to TMAO. The enhancement in the abundance of the *torCAD* operon transcript was sustained into the new TMAO‐respiratory/fermentative steady state but had declined to be only 3.5‐fold greater than that of the initial anaerobic fermentative steady state. Nevertheless, *torCAD* was the only transcript, with the exception of the motility and chemotaxis transcripts discussed below, to exhibit > threefold enhanced abundance in the final TMAO‐respiratory/fermentative steady‐state relative to the initial anaerobic fermentative steady state (Table 2). The pattern of *torCAD* operon transcript abundance is consistent with an initial high expression phase of capacity building during the transition period followed by decreased expression to a level commensurate with the growth rate of the culture in the new TMAO‐respiratory/fermentative steady‐state.

Anaerobic conditions were maintained throughout the experiments described here, and thus it was surprising to find that the transcript encoding a terminal oxidase with low O_2_ affinity (cytochrome *bo*′; Cyo) was strongly induced during the first 10 min of the transition before returning to its initial level of abundance (Table 2; group A). The assertion that the increased abundance of the *cyo* transcript was not caused by unintended introduction of O_2_ was supported by the absence of the broad transcriptional response that is observed upon transfer of *E. coli* cultures to micro‐aerobic or aerobic growth conditions (Partridge *et al*., 2006; Trotter *et al*., 2011; Rolfe *et al*., 2012).

Group B consisted of seven operons involved in carbon metabolism. Like the *cyoA‐E* operon, these were all transiently expressed during the transition to TMAO respiratory growth, returning to their initial abundances in the TMAO respiratory/fermentative steady state (Table 1; group B). These included transcripts encoding key components of central metabolism: pyruvate dehydrogenase, 2‐oxo‐glutarate dehydrogenase and succinate dehydrogenase, indicating that the presence of the electron acceptor TMAO invokes a shift to enhanced substrate oxidation. Also prominent in this grouping were transcripts associated with glycerol and acetate acquisition and metabolism, including both the anaerobic and aerobic glycerol‐3‐phosphate dehydrogenase operons and the glycerol‐3‐phosphate transport system (*ugp* operon assigned to group D). The activation of complex II (succinate dehydrogenase) and the anaerobic and aerobic glycerol‐3‐phosphate dehydrogenases suggests the establishment of respiratory chains with succinate and glycerol‐3‐phosphate as the electron donor in the presence of TMAO. Although extracellular glycerol was not detected in the NMR analysis, the decrease in extracellular succinate concentration and increase in fumarate concentration after addition of TMAO was consistent with the induction of complex II (Table 1). The activation of the *betIBA* operon (glycine‐betaine synthesis from choline; see below) is suggestive of osmotic re‐balancing in the 5 min period after addition of the osmolyte TMAO. A more sustained (> 60 min after TMAO addition) expression profile was observed for the *frmRAB* operon, which encodes a regulator and enzymes involved in formaldehyde detoxification (Table 2; group B).

Group C consisted of operons associated with methionine metabolism. These transcripts were transiently increased in abundance 15–20 min following addition of TMAO to the chemostats before returning to the pre‐TMAO addition level (Table 2; group C). Three of the six transiently induced operons in group C are regulated by MetJ, suggesting that during this period of enhanced biomass production the supply of *S*‐adenosylmethionine, a molecule used in methyl transfer reactions, is restricted (Keseler *et al*., 2013). Group D contained peptide, C_4_‐dicarboxylic acid/orotate, galactose and glycerol‐3‐phosphate transport systems (Table 2; group D).

The transcriptional profile of the new TMAO‐respiratory/fermentative steady state was characterized by enhanced abundance of the flagella and chemotaxis transcripts that formed group E. The abundances of these transcripts did not change during the first 60 min of transition but were increased only in the final TMAO‐respiratory/fermentative steady state (Table 2; group E).

The sixth group consisted of transiently induced transcripts encoding regulatory proteins, proteins of unknown function and proteins involved in osmotic homeostasis (e.g. *betIBA*).

Five operons were transiently downregulated ≥ threefold, with roles in copper resistance, threonine biosynthesis, mannose transport and outer membrane function (Table 2; group G). The decreased abundance of the *cus* operon, which encodes an anaerobic copper detoxification system, suggests that in the initial phases of adaptation the copper stress experienced by the bacteria was diminished.

### Simultaneous inference of TF activities

**Figure 2:**
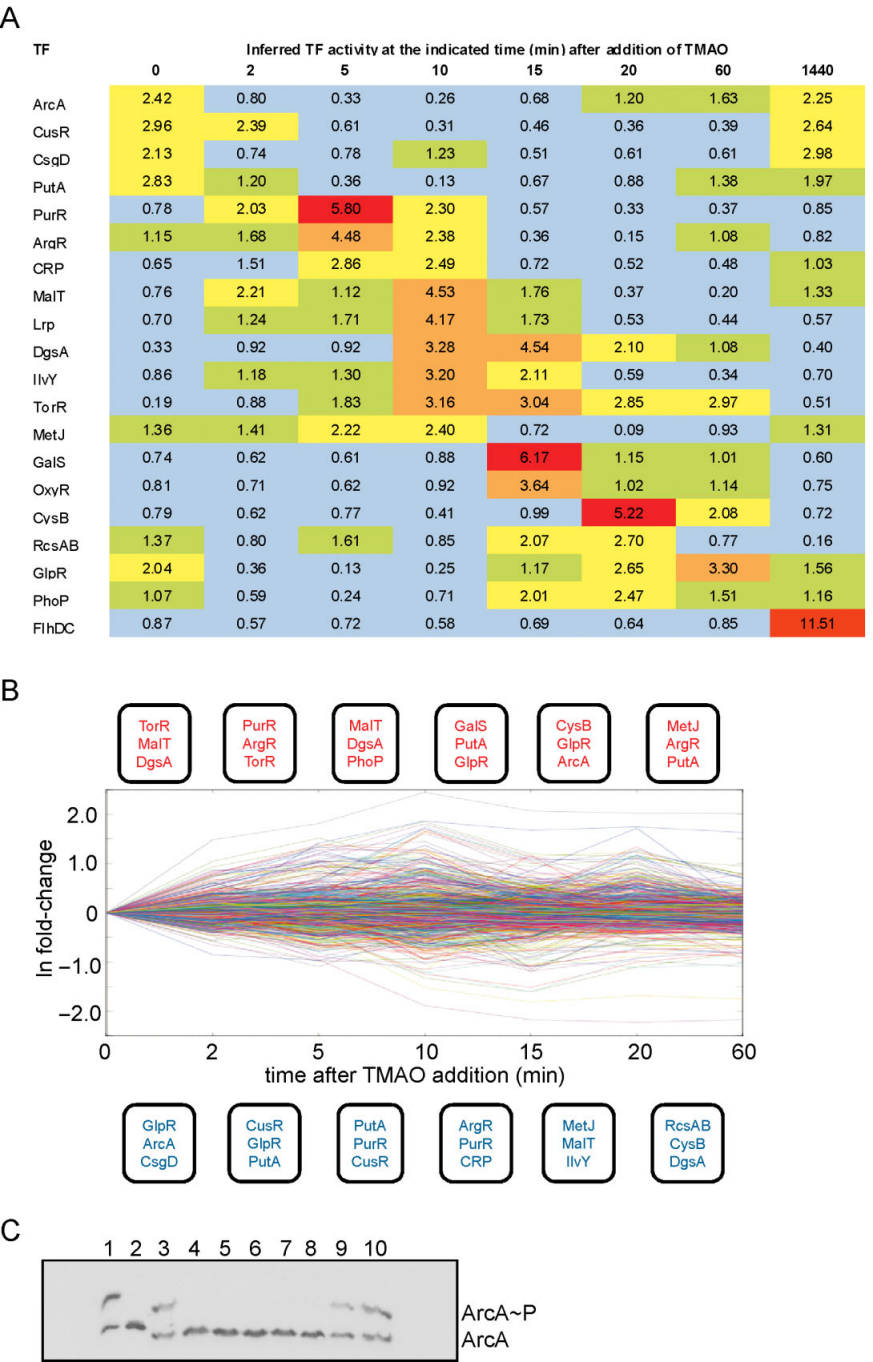
Changes in TF activities in response to perturbation of anaerobic fermentative cultures of *E* *. coli* by switching to TMAO‐respiratory/fermentative metabolism. (A) The output from the tfinfer software (Asif *et al*., 2010) at the indicated times (minutes) after TMAO (46 mM) addition to anaerobic fermentative steady‐state cultures. Each cell is coloured to provide a visual representation of the TF activities inferred from the complete transcript profile dataset as follows: TF activity 0–1, blue; 1–2, green; 2–3, yellow; 3–4, orange; > 4, red. In all cases, the signal to noise ratio was ≥ 2. (B) Graphical representation of regulatory dynamics. The figure shows the time course of all 1381 genes used in the tfinfer analysis; time is re‐scaled to have equal spacing between time points. The panels above and below the time course show the three TFs exhibiting the greatest fold increase (red) or decrease (blue) in activity compared with provious time point as inferred by tfinfer, providing a depiction of the main regulatory events. (C) Validation of inferred ArcA activity by measurement of ArcA∼P. A typical immunoblot of a Phos‐tag gel developed with anti‐ArcA serum. Lane 1, His‐tagged ArcA (1.2 μg) phosphorylated *in vitro* by incubation with carbamoyl phosphate (50 mM); lane 2, His‐tagged dephosphorylated ArcA; lanes 3–10, whole cell samples taken at t = 0, 2, 5, 10, 15, 30, 60 and 1440 min after the addition of TMAO to the anaerobic fermentative steady‐state culture. The locations of ArcA and ArcA∼P are indicated.

The altered patterns of gene expression, substrate utilization and over‐metabolite production described above are the result of complex interactions between signalling molecules and TFs. In addition to the changes in transcript abundance that pass an arbitrary ≥ threefold (*t*‐test, *P* = 0.05) filter (Table 2), there were widespread changes that are components of the transcriptional response that sit below this statistical filter. To capture the breadth of the transcriptional response when the anaerobic fermentative system was perturbed by introduction of the primary signal TMAO, the activities of 167 TFs that are recorded as able to directly regulate target genes in the EcoCyc database (Keseler *et al*., 2013) were simultaneously inferred from the transcript profiles using the open access tfinfer software (Asif *et al*., 2010). tfinfer is based on a state space model that employs a linear approximation (in log space) to the dynamics of transcription and treats noise in a principled way such that the estimated TF activities are associated with confidence limits (Sanguinetti *et al*., 2006). Although the tfinfer model represents a simplified version of transcription, it has proved its utility in several previous studies (e.g. Partridge *et al*., 2007; Rolfe *et al*., 2012). TFs that exhibited a signal to noise ratio > 2 were deemed to have significantly altered activities following the introduction of TMAO (Fig. 2A). The inferred activities of these 20 TFs could account for many of the changes in transcript abundance shown in Table 2 because one or more TFs exhibiting altered activity are known to regulate transcription from the corresponding promoters (Table 2). A visual representation of the regulatory events (as inferred by tfinfer) during the time course is provided in Fig. 2B.

Remarkably, only two TFs (FlhDC and TorR) displayed > twofold enhanced activity in the TMAO‐respiratory steady‐state compared to the initial fermentative steady‐state (Fig. 2A). The explanation for the enhanced FlhDC activity and consequent expression of motility and chemotaxis genes is unclear. However, the inferred activity of TorR implied that an activity ∼sixfold lower than the maximal, which occurred 10 min after introduction of TMAO, is sufficient to maintain an appropriate concentration of TMAO reductase in the new steady state.

Four TFs (ArcA, CusR, CsgD and PutA) exhibited lower activity during the first 60 min of the transition before almost returning to their original activities in the TMAO‐respiratory/fermentative steady state (Fig. 2A). The inferred activities of ArcA were validated by measuring the amount of phosphorylated ArcA (ArcA∼P) present in the bacteria (Fig. 2B). ArcA and ArcA∼P were separated by Phos‐tag gel electrophoresis (Wako Pure Chemical Industries Ltd.) and detected by Western blotting using anti‐ArcA serum. Quantitative densitometry of the blots was used to determine the proportion of ArcA∼P in the samples. This showed that the ArcA∼P decreased from the expected ∼50% level in the anaerobic fermentative steady state (Rolfe *et al*., 2011) to 0% in the 2–20 min period after TMAO addition, before increasing to ∼30% after 60 min and returning to ∼50% in the TMAO‐respiratory/fermentative steady state (Fig. 2B). Thus, direct measurement of the ArcA phosphorylation profile correlated well with the predicted ArcA activities inferred from the transcript profiles. The transient decrease in ArcA activity emphasizes the complexity of the ArcBA response. At least three signals have been proposed as modulators of ArcB activity: oxidized ubiquinone acts negatively; reduced menaquinone acts positively; and fermentation products, such as acetate, promote ArcB kinase activity and ArcA phosphorylation (Georgellis *et al*., 1999; 2001; Rodriguez *et al*., 2004; Bekker *et al*., 2010; Rolfe *et al*., 2011; 2012; Alvarez *et al*., 2013). The dynamics of ArcA activity reported here suggests that introduction of TMAO to the fermenting culture causes net oxidation of the menaquinone pool, resulting in the initial dephosphorylation of ArcA followed by restoration of ArcA activity, in the final TMAO‐respiratory/fermentative steady state, perhaps mediated by enhanced production of acetate (Table 2). The modulation of ArcA activity in this way could account for the surprising transient induction of the cytochrome *bo*′ operon, as well as genes encoding the pyruvate, 2‐oxoglutarate and succinate dehydrogenases because they are all repressed by ArcA (Table 2).

The majority (15 from 20) of responsive TFs (signal to noise ratio ≥ 2), including TorR, exhibited maximal activity during the adaptive phase of the transition before their activities returned to values close to those of the initial steady state. The greatest activities for ArgR, CRP and PurR were 5 min after the introduction of TMAO; IlvY, Lrp, MalT, MetJ and TorR after 10 min; DgsA, GalS and OxyR after 15 min; CysB, RcsAB and PhoP after 20 min; and GlpR after 60 min (Fig. 2A).

### Measurement of TorA protein and TMAO reductase activity suggests that regulation is at the level of transcription

**Figure 3:**
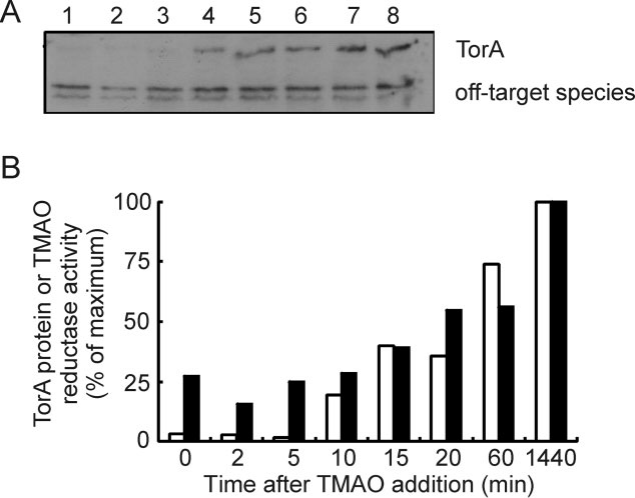
The amount of TorA protein and TMAO reductase activity during adaptation to TMAO‐respiratory growth correlate with the changes in *tor* operon transcription. (A) Typical Western blot of whole cell samples taken from anaerobic fermentative chemostat cultures 0, 2, 5, 10, 15, 30, 60 and 1440 min after the addition of TMAO developed with anti‐TorA serum. (B) Quantitative densitometric analysis of the Western blot shown in A to measure the increase in the amount of TorA protein present in the bacteria after TMAO addition (open bars). The amounts of TorA shown are relative to the level in the final sample (100%). TMAO reductase activities (filled bars) were measured for duplicate samples obtained at the same time points used for quantification of TorA protein (see above). The TMAO reductase activity data are also shown relative to the activity in the final sample (100%).

Measurements of the TorA protein content (Fig. 3A) and TMAO reductase activities (Fig. 3B) after the introduction of TMAO showed that the bacteria grown under fermentative conditions had low TMAO reductase activity, which had increased ∼twofold after 60 min (Fig. 3B). The final ∼fourfold increase in TMAO reductase enzyme activity in the new TMAO‐respiratory/fermentative steady state matched the 3.5‐fold increase in *tor* operon transcript abundance (Table 2), suggesting the absence of post‐transcriptional regulation of TMAO reductase activity. Estimation of the TorA content of the bacteria by Western blotting suggested a greater overall increase in abundance than might be expected based on the increase in TMAO reductase activity, but there was a good positive correlation between enzyme activity and TorA in the sample obtained in the 10–1440 min period of the transition (Fig. 3A and B).

### Transient induction of the formaldehyde dehydrogenase FrmAB is likely to be the result of the presence of TMAO demethylase activity

**Figure 4:**
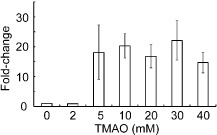
The *frmRAB* operon is induced after exposure of *E* *. coli* MG1655 to concentrations of TMAO ≥ 5 mM. Anaerobic batch cultures were exposed to different concentrations (0–40 mM) of TMAO. After 30 min, total RNA was isolated for qRT‐PCR of the *frmR* mRNA. The data shown and the mean and standard deviation for the fold increase relative to the 0 mM TMAO culture and are typical of three independent experiments.

**Figure 5:**
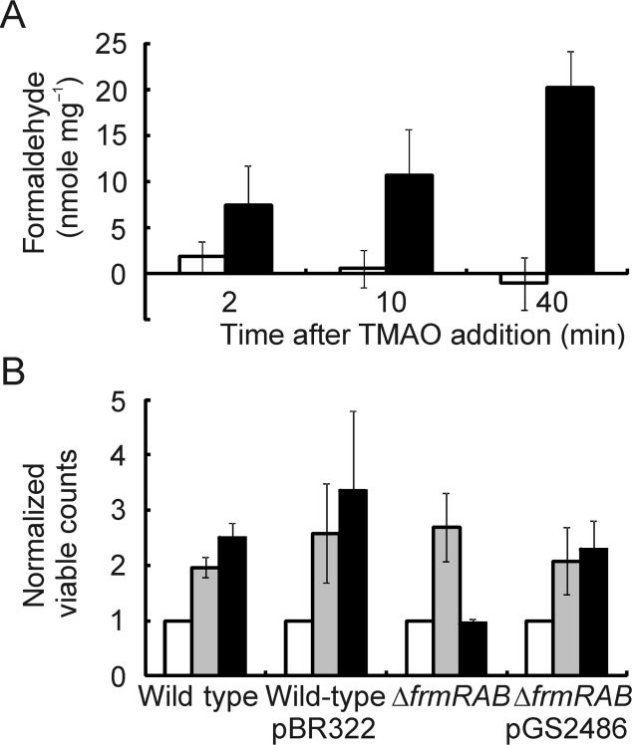
(A) Incubation of *E* *. coli* MG1655 cell‐free extracts with TMAO results in the production of formaldehyde. Cell‐free extracts were incubated with TMAO (40 mM) at 37°C as described in *E* *xperimental procedures*. At the indicated times, formaldehyde production was measured. Open bars, cell‐free extract only; closed bars, cell‐free extract plus TMAO. The data shown are the mean values ± standard deviation obtained from three independent experiments. (B) The *E* *. coli* MG1655 *frmRAB* mutant is attenuated when exposed to TMAO. Anaerobic cultures of *E* *. coli* MG1655 (wild type), wild type transformed with the vector pBR322, the *frmRAB* mutant and the mutant complemented with the *frmRAB* expression plasmid pGS2486 were grown on glucose minimal medium buffered with 50 mM phosphate buffer, pH 7.0, at 37°C. In an anaerobic cabinet, each culture divided into two separate cultures and samples for viable count measurements were taken (t = 0 min; white bars). TMAO (40 mM) was added to one (black bars), but not the other (grey bars), of each pair of cultures and anaerobic incubation was continued for a further 60 min, at which point samples for viable count measurements were taken. The data shown are the means and standard deviations normalized to the values obtained at t = 0 min obtained from three independent biological replicates.

The *frmRAB* operon is induced by formaldehyde, and its expression is negatively regulated by FrmR, encoded by the first gene of the operon (Herring and Blattner, 2004). FrmA and FrmB proteins catalyse the glutathione‐dependent conversion of formaldehyde to formate (Gutheil *et al*., 1992). Thus, the transient increased abundance of the *frmRAB* transcript upon exposure of the cultures to TMAO was unexpected (Table 2). Therefore, confirmation of *frmRAB* induction by TMAO was sought by quantitative reverse transcription polymerase chain reaction (qRT‐PCR) analyses of RNA isolated from batch cultures of *E. coli* MG1655 after 60 min exposure to different concentrations (0–40 mM) of TMAO. The data showed that the abundance of the *frmRAB* transcript was significantly increased when the bacteria were exposed to ≥ 5 mM TMAO (Fig. 4). Hence, these experiments confirmed the transcriptional response detected by the transcript profiling and showed that this adaptation occurred in both continuous and batch cultures and that *frmRAB* induction occurred at TMAO concentrations ≥ 5 mM (Table 2; Fig. 4). Incubation of *E. coli* K‐12 cell‐free extracts with TMAO (40 mM) allowed the detection of formaldehyde using the Nash reagent (Fig. 5A). Control reactions lacking cell‐free extract or TMAO failed to accumulate formaldehyde. Furthermore, closer inspection of the extracellular metabolite profile of the chemostat cultures revealed the presence of up to 0.23 mM dimethylamine (DMA) after the addition of TMAO (Table 1). These observations suggest that *E. coli* K‐12 possesses TMAO demethylase activity, resulting in the formation of sub‐millimolar DMA and formaldehyde, the latter accounting for the induction of *frmRAB* expression, which is known to be induced when *E. coli* cultures are exposed to 0.25 mM formaldehyde (Herring and Blattner, 2004); a concentration very similar to the amount of DMA detected in the cultures analysed here (Table 1).

The detection of formaldehyde when cell‐free extracts were incubated with TMAO and the induction of the *frmRAB* operon during transition to TMAO respiratory growth suggested that the inability of an *frmRAB* mutant to detoxify formaldehyde would compromise its fitness when exposed to TMAO. Therefore, an isogenic *E. coli* MG1655 *frmRAB* mutant (JRG6703) was created along with a plasmid (pGS2486) to express *frmRAB* under the control of its own promoter to complement the mutant. The numbers of colony‐forming units (cfu) present in anaerobic cultures of the parent, mutant and complemented mutant before and after incubation with TMAO were measured. The results showed that the cfu values of the parent and complemented mutant cultures increased after 60 min exposure to 40 mM TMAO, but the cfu value of the *frmRAB* mutant did not (Fig. 5B). This result is consistent with production of potentially toxic concentrations of formaldehyde, which requires a regulatory response to prevent inhibition of growth during the transition to anaerobic respiration with TMAO as the terminal electron acceptor.

### TMAO/TMAO reductase competes with Cu(II) for electrons from the anaerobic electron transport chain

**Figure 6:**
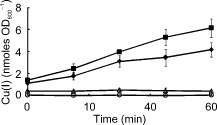
Effect of TMAO on Cu(II) reduction by *E* *. coli* MG1655. Bacterial cell suspensions (final OD_600_ ∼ 2.5) in Evan's medium were incubated at 37°C for up to 60 min in the presence and absence of CuSO_4_ (0.05 mM) and/or TMAO (40 mM). At the indicated time points, aliquots were removed and the amount of Cu(I) present was measured. Open circles, no bacteria; open triangles, no Cu(II); closed diamonds, bacteria plus Cu(II) plus TMAO; closed squares, bacteria plus Cu(II). The data are the mean values ± standard deviation obtained from three independent experiments.

**Figure 7:**
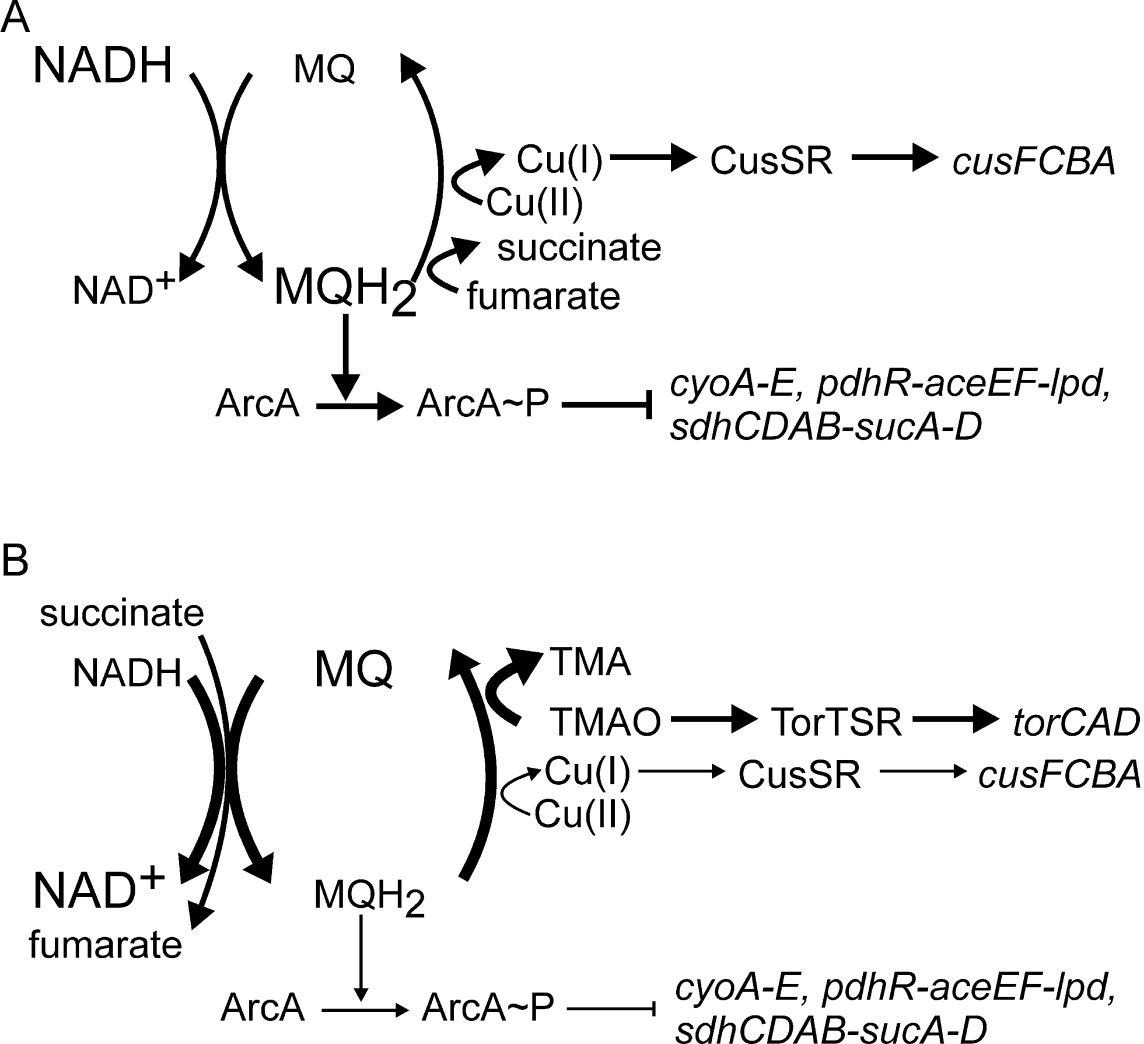
Excess TMAO activates TorTSR, inhibits Cu(II) reduction by the electron transport chain and permits activation of ArcA. (A) In the absence of a non‐endogenous electron acceptor (anaerobic fermentation), fumarate is reduced to succinate in the reductive arm of the anaerobic Krebs cycle and the trace element Cu(II) is reduced to Cu(I) both at the expense of menaquinol (MQH_2_). The Cu(I) generated by this process is sensed by the CusSR two‐component system, and the copper efflux system CusFCBA is activated. Flux through the electron transport chain is relatively low, and the MQH_2_ : MQ ratio is high resulting in the phosphorylation of ArcA (ArcA∼P) and repression of the *cyoA‐E*, *pdhR‐aceEF‐lpd* and *sdhCDAB‐sucA‐D* operons. (B) During the acute phase of adaptation to the presence of excess TMAO is sensed by the TorTSR sensor‐regulator system, which activates expression of the *torCAD* operon encoding TMAO reductase. TMAO reductase effectively competes for the electrons of the menoquinone pool such that the MQH_2_ : MQ ratio is low, which decreases phosphorylation of ArcA resulting in de‐repression of the *cyoA‐E*, *pdhR‐aceEF‐lpd* and *sdhCDAB‐sucA‐D* operons. This could permit the establishment of a respiratory chain with succinate as the electron donor and TMAO as the electron acceptor, consistent with the observed transcriptional profile and the concentrations of succinate and fumarate in the culture medium. The flux of electrons to TMAO lowers the capacity of the bacteria to reduce Cu(II) to Cu(I), and hence the TF CusR is switched off. The width of the regulatory arrows is an indication of the relative rate of each step, and font size is used to indicate the NADH : NAD ^+^ and MQH_2_ : MQ ratios.

The inferred decrease in activity of the TF CusR implied that after introduction of TMAO, the exposure of the *E. coli* cultures to Cu(I) was diminished because the signal for the cognate sensor CusS is Cu(I). The trace element Cu(II) can be reduced to Cu(I) (E_m_ + 340 mV) by *E. coli* using electrons transferred to ubiquinone by NADH dehydrogenase II (E_m_ NAD^+^/NADH, −320 mV; ubiquinone/ubiquinol + 110 mV) under aerobic conditions (Thauer *et al*., 1977; Volentini *et al*., 2011). Under anaerobic conditions, menaquinone (E_m_ menaquinone/menaquinol, −74 mV) is the electron donor for the TMAO reductase (Wissenbach *et al*., 1992) but like ubiquinone has the potential to reduce Cu(II). Therefore, the capacity of *E. coli* K‐12 MG1655 to reduce Cu(II) was tested under anaerobic conditions in the presence and absence of TMAO. Under both conditions Cu(II) reduction was detected, but this was diminished ∼1.6‐fold in the presence of TMAO (Fig. 6). In combination with the data described above, the Cu(II) reduction experiments suggest that under anaerobic fermentative conditions there is a relatively low flux of electrons from the menaquinone pool to fumarate reductase to reduce fumarate to succinate (Table 1), allowing electrons to leak directly or indirectly from reduced quinones to periplasmic Cu(II) resulting in the production of Cu(I) and activation of the CusSR transcriptional response (Table 2; Fig. 7A). Supplying the electron acceptor TMAO to the culture offers an additional outlet for the electrons in the menaquinone pool resulting in decreased reduction of Cu(II) to Cu(I) by the electron transport chain during the initial adaptation phase (Fig. 6; Fig. 7B). In this way, CusR is inactivated resulting in decreased abundance of the *cus* operon transcript (Fig. 2A; Table 2). Furthermore, a shift towards a more oxidized menaquinone pool in the presence of excess TMAO would contribute to the inactivation of ArcA and hence de‐repression of the *cyoA‐E*, *pdhR‐aceEF‐lpd* and *sdhCDAB‐sucA‐D* operons (see above; Fig. 2, Table 2).

### Conclusion

The experiments described above have provided new insights into the dynamic adaptive processes that occur when *E. coli* switches metabolic mode from anaerobic fermentative growth to the energetically more efficient anaerobic respiratory growth with TMAO as the terminal electron acceptor (Fig. 7). As well as predictable changes in the transcript and metabolite profiles, such as the induction of the *torCAD* operon and the reduction of TMAO to TMA, some unexpected components of the adaptive process were revealed. It will be of interest to perform similar experiments with mutants lacking some of the key regulators (e.g. ArcA, FlhDC, FrmR and MetJ) identified here to further characterize the roles played by their respective regulons in adaptation to TMAO exposure.

The detection of DMA and formaldehyde suggests that *E. coli* possesses TMAO demethylase activity that produces physiologically significant amounts of formaldehyde in environments in which TMAO is abundant. The formaldehyde generated by this activity is sufficient to induce the expression of the formaldehyde detoxifying FrmAB enzyme when TMAO is in excess. Work is ongoing to attempt to identify the source of the TMAO demethylase activity. The transient inactivation of ArcA predicted by probabilistic modelling of the transcript profiling data was validated by direct measurement of ArcA∼P and provided an explanation for the surprising induction of the *cyoA‐E* transcript that encodes a low O_2_‐affinity terminal oxidase despite maintaining anaerobic culture conditions throughout the experiments. In addition to *cyoA‐E*, the induction of several other ArcA∼P‐repressed operons could be accounted for by the temporary inactivation of ArcA. As stated above, the ArcBA two‐component system acts as an indirect sensor of O_2_ by ArcB monitoring the redox state of the electron transport chain and the production of fermentation products. Thus, ArcBA activity is sensitive to cellular NADH/NAD^+^ ratios (Holm *et al*., 2010). It has been shown that glucose‐limited chemostat cultures of *E. coli* are affected by the availability of electron acceptors fumarate, nitrate and oxygen, which act as effective NADH sinks. The greatest NADH/NAD^+^ ratios (∼0.75) were observed during anaerobic fermentative growth, and lower ratios were measured when an external environmental electron acceptor (∼0.3 with 7.5 mM nitrate; ∼0.4 with 70 mM fumarate; and ∼0.08 under fully aerobic conditions) was available (de Graef *et al*., 1999; Alexeeva, 2000). Addition of excess TMAO to the anaerobic fermentative culture would be expected to result in a decreased NADH/NAD^+^ ratio by providing an outlet for the electrons held in the menaquinone pool during the initial phase of adaptation, resulting in transient dampening of ArcBA activity, which is then counterbalanced by increased production of acetate as TMAO‐respiratory metabolism is established. Competition for electrons from the menaquinone pool could also account for the downregulation of copper resistance genes in the presence of excess TMAO. Previous work has shown that electrons from the *E. coli* respiratory chain can reduce Cu(II) to Cu(I) (Volentini *et al*., 2011). The demonstration that TMAO was able to inhibit reduction of Cu(II) by *E. coli* suggests that TMAO reductase/TMAO are effective competitors for electrons that might otherwise have contributed to the reduction of Cu(II). The lower production of Cu(I) would result in the inactivation of CusR and downregulation of copper resistance genes. Thus, systematic analysis of *E. coli* cultures has provided new insights into the dynamics of transcriptional and physiological changes required to successfully adapt to anaerobic environments containing TMAO.

## Experimental procedures

### Bacterial strains and growth conditions

*Escherichia coli* K‐12 MG1655 and the isogenic *frmRAB* mutant (JRG6703 created using λred recombineering; Datsenko and Wanner, 2000), along with strains transformed with either the plasmid pGS2486 to express *frmRAB* under the control of its own promoter or the corresponding vector control (pBR322), were used in this study.

Glucose‐limited steady‐state continuous cultures of *E. coli* MG1655 were established in a 2 l Labfors chemostat (Infors‐HT, Bottmingen, Switzerland) at 37°C, a 950 ml working volume, a stirring rate of 400 r.p.m., a dilution rate of 0.2 h and a gas flow rate of 1 l min^−1^ in Evans medium (10 mM NaH_2_PO_4_, 10 mM KCl, 1.25 mM MgCl_2_, 100 mM NH_4_Cl, 2 mM Na_2_SO_4_, 2 mM nitrilotriacetic acid, 0.02 mM CaCl_2_, 25 μM ZnO, 10 μM FeCl_3_, 50 μM MnCl_2_, 5 μM CuCl_2_, 11 μM CoCl_2_, 0.05 μM H_3_BO_3_, 0.08 μM Na_2_MoO_4_) supplemented with 30 μg l^−1^ sodium selenite, and 20 mM glucose as a carbon source (Evans *et al*., 1970). Anaerobic conditions were maintained by sparging with 95% N_2_ and 5% CO_2_ gas. The pH was maintained at 6.9 by titration with 1 M NaOH. Steady‐state fermentative cultures were perturbed by addition of 45.8 ± 1.5 mM TMAO to the medium feed and directly into the chemostat. Samples were removed from the chemostat at the stated times (in minutes) after the addition of TMAO.

For some experiments, *E. coli* strains were grown as batch cultures in rich medium [Luria–Bertani (LB) – yeast extract, 5 g l^−1^; tryptone, 10 g l^−1^; NaCl, 5 g l^−1^] with supplements as indicated.

### NMR metabolite measurements

Extracellular carbon metabolite concentrations were determined as described previously (Rolfe *et al*., 2011; Trotter *et al*., 2011). Bacteria were removed by passing chemostat culture samples through 0.22 μm pore size filters. The cell‐free samples were analysed in 5 mm NMR tubes containing 450 μl supernatant, 50 μl D_2_O and 1 mM (final concentration) trimethylsilyl propanoic acid (TSP) as an internal standard. Spectra were acquired on a Bruker DRX500 spectrometer (Bruker UK) at 298 K with 5 mm TXI probe, tuned to ^1^H at 500 MHz. The H_2_O signal was reduced by pre‐saturation for 2 s applied during the recycle time while carbon decoupling was applied during acquisition to suppress ^13^C satellites. Spectra were processed and peaks quantified by integration using Topspin (Bruker UK) while chemical shifts and metabolite concentrations were established by reference to the TSP peak.

### Transcriptional profiling

Transcriptional profiling was carried out in a reference style (Rolfe *et al*., 2011) for three biological replicates. Briefly, samples were removed from the chemostat directly into RNA protect (Qiagen) before purification of total RNA using the RNeasy mini kit (Qiagen). Genomic DNA (2.0 μg) was converted to Cy5‐labelled cDNA, and purified RNA (16 μg) (from each time point) was converted to Cy3‐labelled cDNA. These were combined and hybridized overnight to an oligonucleotide microarray obtained from Agilent Technologies (AMADID 029412). Quantification of cDNA samples, hybridization to microarrays, microarray processing and scanning were carried out as described in the Fairplay III labelling kits (Agilent Technologies, 252009, Version 1.1) and scanned with a high‐resolution microarray scanner (Agilent Technologies). genespring gx v7.3 (Agilent Technologies) was used for data normalization and analysis. Transcripts exhibiting > threefold change in abundance after *t*‐test with a *P*‐value of < 0.05 at one or more of the time points analysed were deemed to be differentially regulated. The data are available in ArrayExpress E‐MTAB‐2784. The relative activities of TFs were inferred using the software package tfinfer as previously described (Asif *et al*., 2010).

### qRT‐PCR

Anaerobic cultures were grown overnight at 37°C in anaerobic Evans medium buffered with 50 mM phosphate buffer, pH 7.0, and supplemented with 20 mM glucose. The amounts of *frmR* mRNA in samples of total RNA (100 ng; isolated as described above) after exposure of these cultures to different concentrations of TMAO for 30 min were determined on an RT‐PCR plate in a Mx3005P Thermocycler (Agilent Technologies) using Brilliant III Ultra‐Fast SYBR Green qRT‐PCR Master Mix kit (Agilent Technologies) according to the manufacturer's instructions. The housekeeping gene *gyrA* was used for normalization, and a genomic DNA dilution series was used to correct for differences in primer amplification efficiencies. The sequences of the primer sets were: *frmR* CTGGAACGGTCGCTGGAG, ATCAGCCCATTAGCCGCG; *gyr*A ACCTTGCGAGAGAAATTACACC, AATGACCGACATCGCATAATC.

### Measurement of ArcA phosphorylation

The degree of ArcA phosphorylation was measured using Phos‐tag‐acrylamide gel electrophoresis and subsequent Western immunoblotting. Chemostat culture samples were collected directly into a final concentration of 1 M formic acid as previously described (Rolfe *et al*., 2011). Samples were then processed as described elsewhere (Barbieri and Stock, 2008) using OD_600_ measurements to calculate volumes of sample to be resolved on a 37.5 μM Phos‐tag‐10% acrylamide gel. Standard wet transfer to a Hybond‐C membrane (Amersham) was followed by immunodetection using rabbit ArcA antiserum and the ECL detection system (GE Healthcare). ArcA protein was purified and phosphorylated *in vitro* as previously described (Bekker *et al*., 2010) with the exception of using 100 mM carbamoyl phosphate.

### Detection of TorA protein by Western immunoblotting

Chemostat culture samples were collected directly into a final concentration of 50 mg ml^−1^ chloramphenicol. Aliquots of the suspension were pelleted by centrifugation (10 000 r.p.m. in a microcentrifuge for 3 min) and pellets stored at −80°C until required. Samples were standardized with respect to their OD_600_ value by dilution with water. The samples were boiled in SDS‐PAGE loading buffer for 15 min prior to being resolved on a 10% acrylamide gel followed by electroblotting onto Hybond‐C membrane (Amersham). TorA protein was detected using rabbit TorA antiserum (1:1000) and visualized using the ECL system (GE Healthcare) according to manufacturer's instructions.

### TMAO reductase assays

Methyl viologen‐linked reductase assays were carried out with intact cell samples from the chemostat. Cell samples were washed and re‐suspended in 25 mM sodium phosphate buffer pH 7.0 to OD_600_ ∼6. Reductase assay measurements were carried out as previously described (Hitchcock *et al*., 2010), except 0.2 mM methyl viologen was used. Duplicate assays were carried out in screw‐topped glass cuvettes fitted with silicon seals in a total volume of 1 ml in a CARY 50 UV‐visible spectrophotometer (Agilent Technologies) pre‐warmed to 37°C. All buffers and reagents were made anaerobic prior to the assay by sparging with argon. TMAO reductase activities were expressed relative to OD_600_ of the intact cell sample.

### Measurement of formaldehyde production

Formaldehyde was measured using acetylacetone reagent (Nash, 1953). Preparation of cell samples and setting up of assays were performed in an anaerobic workstation. Anaerobic cultures of *E. coli* MG1655 were grown overnight with stirring at 37°C in LB supplemented with 0.2% glucose. Cells were pelleted, washed in 25 mM sodium phosphate buffer pH 7.0 and re‐suspended in a final volume of 2 ml anaerobic cell breakage buffer. An oxygen scavenger (10% glucose, 10 μg ml^−1^ catalase and 5 μg ml^−1^ glucose oxidase), 1 ml, was added to the re‐suspended cells before sonication (6 × 15 s, 15 micron amplitude). Any unbroken cells were removed by centrifugation at 5500 r.p.m. in a benchtop microcentrifuge for 1 min. Cell‐free extracts were added to a 1 ml total volume of 25 mM sodium phosphate buffer and 40 mM TMAO (in TMAO‐containing samples only). Assays were incubated anaerobically at 37°C and at the indicated times were stopped by precipitation using 5% trichloroacetic acid and centrifugation. Resulting supernatants were treated as previously described (Fu *et al*., 2006). Formaldehyde content was calculated using the extinction coefficient at 412 nm (8000 M cm^−1^). Protein content of cell‐free extracts was determined using the Bio‐Rad assay.

### Cell viability in the presence of TMAO

Anaerobic cultures were grown overnight at 37°C in anaerobic Evans medium buffered with 50 mM phosphate buffer, pH 7.0, and supplemented with 20 mM glucose in 7 ml bijoux. After 18 h growth, a 10% inoculum of the culture was added to 7 ml Evans medium in an anaerobic workstation, and pre‐treatment viable counts were measured by serial dilution in medium. The 7 ml culture was split in half (2 × 3.5 ml), and 40 mM TMAO was added to one of the subcultures. The cultures were incubated in parallel for a further 1 h at 37°C before post‐treatment viable counts were measured. Plated cells were grown overnight at 37°C on LB agar and cfu per millilitre calculated.

### Copper (II) reduction assays

*Escherichia coli* MG1655 cells were grown anaerobically overnight at 37°C in Evans medium (pH 7.0) supplemented with 20 mM glucose and 40 mM TMAO. After 18 h, cells were pelleted and washed three times in anaerobic Evans medium, pH 7.0. Cells were re‐suspended in Evans medium plus 20 mM glucose to an OD_600_ ∼25. The assays were prepared as previously described (Volentini *et al*., 2011) in an anaerobic workstation. Briefly, 100 μl of washed cells were incubated with 0.05 mM CuSO_4_, 40 mM TMAO (in TMAO‐containing samples) in a 1 ml total volume of citrate phosphate buffer, pH 7.0, at 37°C. Control assays with no cells and no CuSO_4_ were carried out in parallel. At the indicated times, the assays were stopped by pelleting the cells and 0.1 mM bathocuproine disulfonate (Sigma‐Aldrich) was added to the resulting supernatant. The absorbance at 412 nm for the supernatant was measured and the molar extinction coefficient for the Cu(I) complex (12 220 M cm^−1^) used to determine Cu (II) reduction by the samples per OD_600_ unit.
